# Supplementary material for: ‘It takes two to tango’: Bridging the gap between country need and vaccine product innovation
Source: PLoS One. 2020 Jun 10;15(6):e0233950. doi: 10.1371/journal.pone.0233950 (PMC7286512; doi:10.1371/journal.pone.0233950)
Supplement: S1 Table — (DOCX) [file pone.0233950.s001.docx]

**S1 Table. Vaccine characteristics of the five hypothetical rotavirus vaccine products**

| **Vaccine characteristics** | **Rotavirus vaccine products*** | | | | |
| --- | --- | --- | --- | --- | --- |
|  | **RVV-1** | **RVV-2** | **RVV-3** | **RVV-4** | **RVV-5** |
| Number of doses needed | 3 | 2 | 1 | 3 | 3 |
| Vaccine efficacy | 50% | 50% | 65% | 60% | 72% |
| Duration of protection (weeks) | 52 | 52 | 52 | 78 | 52 |
| Dosing schedule of first dose | DTP-1 | DTP-1 | DTP-1 | OPV-1 | DTP-1 |
| Price per dose (USD) | 3.6 | 2.2 | 5 | 8.2 | 6.1 |
| Doses per vial | 1 | 1 | 1 | 2 | 1 |
| Volume of the vaccine per dose (cm^3^) | 46.3 | 17.6 | 24.3 | 22.1 | 34.2 |
| Does it need a diluent? | Yes | Yes | No | No | Yes |
| Volume of diluent/ other component (cm^3^) | 45 | 17.6 | - | - | 42 |
| Is an injection syringe/ applicator required? | No | Yes | No | No | Yes |
| Volume of injection syringe (cm^3^) | - | 2 | - | - | 12 |
| Price of injection syringe/ applicator (USD) | - | 0.04 | - | - | 0.02 |
| Is a reconstitution syringe/adapter required? | No | No | No | No | Yes |
| Volume of reconstitution syringe/adapter (cm^3^) | - | - | - | - | 21 |
| Price of reconstitution syringe/ applicator | - | - | - | - | 0.01 |
| Method of cooling, Vaccine |  |  |  |  |  |
| National level | Cold chain | Cold chain | CTC | Cold chain | Freezer |
| Regional level | Cold chain | Cold chain | CTC | Cold chain | Freezer |
| District level | Cold chain | Cold chain | CTC | Cold chain | Freezer |
| Health centre level | Cold chain | Cold chain | CTC | Cold chain | Freezer |
| Method of cooling, Diluent |  |  |  |  |  |
| National level | Cold chain | Cold chain | - | - | Freezer |
| Regional level | Cold chain | Cold chain | - | - | Freezer |
| District level | Cold chain | Cold chain | - | - | Freezer |
| Health centre level | Cold chain | Cold chain | - | - | Freezer |
| Relative risk of a intussusception adverse event in the 1-7 days risk period | | | | | |
| Dose 1 | 6 | 4.2 | 3 | 2 | 3 |
| Dose 2 | 3 | 2 | - | 1 | 1 |
| Dose 3 | 1.7 | - | - | 1 | 1 |
| Relative risk of a intussusception adverse event in the 8-21 days risk period | | | | | |
| Dose 1 | 1.5 | 1.4 | 0 | 1.2 | 1.1 |
| Dose 2 | 1 | 1 | - | 1 | 1 |
| Dose 3 | 1 | - | - | 1 | 1 |
| Wastage | 5% | 5% | 5% | 5% | 5% |
| Buffer vaccines | 10% | 10% | 10% | 10% | 10% |
| *All five vaccine products considered are hypothetical | | | | | |
| cm^3^, cubic centimetre; CTC, controlled temperature chain; DTP, a combined vaccine against diphtheria, tetanus, and pertussis; OPV, oral polio vaccine; RVV, rotavirus vaccine; USD, United States Dollar | | | | | |
