# Supplementary material for: ‘It takes two to tango’: Bridging the gap between country need and vaccine product innovation
Source: PLoS One. 2020 Jun 10;15(6):e0233950. doi: 10.1371/journal.pone.0233950 (PMC7286512; doi:10.1371/journal.pone.0233950)
Supplement: S2 Table — (DOCX) [file pone.0233950.s002.docx]

| **Variables** | **Description** |
| --- | --- |
| Size of target population | Size of birth cohort |
| Coverage of existing vaccines for different population segments | Coverage of existing OPV-1/ DTP1-3 vaccines in Thailand by wealth quintiles and administrative regions |
| Vaccine schedules with which rotavirus is expected to be administered | Schedule of OPV-1 and DTP 1,2,3 vaccine programs |
| Mortality rates | Background mortality, infant mortality and neonatal mortality |
| Disease incidence and related statistics | Incidence of rotavirus infection by severity levels and related mortality |
| Use of healthcare services | Distribution of inpatient and outpatient visits by healthcare setting - primary, secondary, tertiary and healthcare centres |
| Healthcare costs | Healthcare costs for outpatient and inpatient cases by health care setting – primary, secondary, tertiary and healthcare centres |
| Adverse events incidence | Background intussusception rate per 100,000 |
| Vaccine storage and delivery | Thailand specific costs for vaccine transport and storage |
| DTP, a combined vaccine against diphtheria, tetanus, and pertussis; OPV, oral polio vaccine | |

**S2 Table. Input variables required for populating the**

**TSE model**
