# Supplementary material for: ‘It takes two to tango’: Bridging the gap between country need and vaccine product innovation
Source: PLoS One. 2020 Jun 10;15(6):e0233950. doi: 10.1371/journal.pone.0233950 (PMC7286512; doi:10.1371/journal.pone.0233950)
Supplement: S3 Table — (DOCX) [file pone.0233950.s003.docx]

**S3 Table. Vaccine characteristics variables for the vaccine RVV-2 used in sensitivity analysis**

| **Vaccine Characteristics** | **Base case** | | **Best case** | |
| --- | --- | --- | --- | --- |
|  | **Input Values** | **Sources/ Rationale** | **Input Values** | **Sources/ Rationale** |
| Relative risk of intussusception | 1.24 | As per the initial values assumed in base case which can be found in Supplementary Table 2. | 1.0 | RR of 1 represents that there is no increase in risk of intussusception due to the vaccine as compared to the annual risk without vaccination |
| Number of doses | 2 |  | 1 | A single dose vaccine is the best case as the infant gets full protection for the vaccine by only a single dose. This increases the overall adherence and vaccine efficacy in infants |
| Vaccine Schedule | DTP-1 (6 weeks after birth) |  | OPV-1 (1 week after birth) | Administration of the rotavirus vaccine with the OPV-1 vaccine is the best case, as it is reported to have a higher compliance among the population in Thailand. |
| Vaccine efficacy | 50% |  | 100% | The best case assumes the vaccine to have perfect efficacy |
| Duration of Protection (weeks) | 52 |  | 156 | The best case represents the vaccine to have 3 times longer duration of protection as compared to base case |
| Commodity cost (US$) | 2.2 |  | 1.1 | The best case assumes the vaccine cost to be half of its initial cost in the base case |
| Volume of the vaccine (m^3^) | 17.6 |  | 8.8 | The best case assumes the volume of the vaccine to be half of the volume assumed in the base case |
|  | | | | |
