# Supplementary material for: ‘It takes two to tango’: Bridging the gap between country need and vaccine product innovation
Source: PLoS One. 2020 Jun 10;15(6):e0233950. doi: 10.1371/journal.pone.0233950 (PMC7286512; doi:10.1371/journal.pone.0233950)
Supplement: S4 Table — (DOCX) [file pone.0233950.s004.docx]

**S4 Table. Scores and ranks for the five hypothetical rotavirus vaccine products using the TSE approach**

| **Absolute scores** | | | | | | |
| --- | --- | --- | --- | --- | --- | --- |
| **Criteria** | | RVV1 | RVV2 | RVV3 | RVV4 | RVV5 |
| Safety | | 0 | 42 | 77 | 83 | 74 |
| Health impact | | 17 | 17 | 23 | 34 | 24 |
| Budget impact | | 54 | 85 | 87 | 0 | 26 |
| Delivery costs | | 7 | 79 | 98 | 65 | 0 |
| Cost-effectiveness | | 0 | 61 | 27 | 0 | 0 |
| **Weighted scores** | | | | | | |
| **Criteria** | **Weights** | RVV1 | RVV2 | RVV3 | RVV4 | RVV5 |
| Safety | 20% | 0 | 8 | 15 | 17 | 15 |
| Health impact | 20% | 3 | 3 | 5 | 7 | 5 |
| Budget impact | 20% | 11 | 17 | 17 | 0 | 5 |
| Delivery costs | 20% | 8 | 17 | 20 | 15 | 0 |
| Cost-effectiveness | 20% | 0 | 12 | 15 | 0 | 0 |
| Aggregate weighted score | | 22 | 58 | 62 | 39 | 25 |
| **Vaccine ranking** | | **5^th^** | **2^nd^** | **1^st^** | **3^rd^** | **4^th^** |
| RVV, rotavirus vaccine | | | | | | |
