# Supplementary material for: ‘It takes two to tango’: Bridging the gap between country need and vaccine product innovation
Source: PLoS One. 2020 Jun 10;15(6):e0233950. doi: 10.1371/journal.pone.0233950 (PMC7286512; doi:10.1371/journal.pone.0233950)
Supplement: S5 Table — (DOCX) [file pone.0233950.s005.docx]

**S5 Table. Scores and ranks for RVV-2, the second ranking vaccine product, in the base case and best case sensitivity analysis**

The below table shows the scores for the vaccine RVV-3 in base case and RVV-3 for the base case and 7 other best case scenario for RVV-2. The left column lists the vaccine characteristic value assumed in the best case, with all the other characteristics similar to its base case value. In base case, the weighted score of RVV-3 is greater than the score for RVV-2 and hence it is the top ranked vaccine. In the different best case scenario we notice that the overall score of RVV-2 becomes greater than RVV-3 on varying many variable characteristics, making RVV-2 as the top ranked vaccine product.

|  | **Decision Criteria** | | | | | |
| --- | --- | --- | --- | --- | --- | --- |
| ***Best case scenario by vaccine characteristics*** | **Safety** | **Health Impact** | **Budget Impact** | **Delivery Costs** | **Cost-effectiveness** | **Weighted Score*** |
| Base case scores RVV-3 | 77 | 23 | 87 | 98 | 27 | **62** |
| Base case scores for RVV-3 | 42 | 17 | 85 | 79 | 61 | 58 |
| ***Scores for RVV-2 for best case scenarios*** | | | | | | |
| Relative risk of intussusception = 1 | 100 | 17 | 86 | 79 | 64 | **69** |
| Number of doses = 1 | 63 | 18 | 97 | 79 | 94 | **70** |
| Vaccine Schedule= OPV | 41 | 18 | 85 | 79 | 62 | 57 |
| Vaccine efficacy = 100% | 42 | 35 | 94 | 79 | 94 | **69** |
| Duration of Protection (weeks) = 156 | 42 | 42 | 95 | 79 | 98 | **71** |
| Commodity cost (US$) = 1.1 | 42 | 17 | 96 | 79 | 92 | **65** |
| Volume of the vaccine (m^3^) = 8.8 | 42 | 17 | 86 | 94 | 62 | 60 |
| *^*^Assuming weights for all criteria as 20%* | | | | | | |
