# Supplementary material for: ‘It takes two to tango’: Bridging the gap between country need and vaccine product innovation
Source: PLoS One. 2020 Jun 10;15(6):e0233950. doi: 10.1371/journal.pone.0233950 (PMC7286512; doi:10.1371/journal.pone.0233950)
Supplement: S6 Table — (DOCX) [file pone.0233950.s006.docx]

**S6 Table. Thematic analysis results from self-assessment forms**

| **Themes** | **Codes** |
| --- | --- |
| Potential to strengthen product selection progress | Enhanced dialogue between stakeholders |
|  | Products responsive to country needs |
|  | Promote evidence-informed decision making |
|  | Efficient research and development process |
| Limited local capacity | Scarce data |
|  | Low research and development capacity |
|  | Limited technical expertise |
|  | Weak surveillance |
|  | Limited HTA infrastructure |
| Technical assistance | Demonstrate the value-added |
|  | Provider training |
|  | Establish guidelines |
| Concerns of applicability | User of TSE unclear |
|  | Lack of flexibility |
|  | Requires political commitment |
