# Supplementary material for: ‘It takes two to tango’: Bridging the gap between country need and vaccine product innovation
Source: PLoS One. 2020 Jun 10;15(6):e0233950. doi: 10.1371/journal.pone.0233950 (PMC7286512; doi:10.1371/journal.pone.0233950)
Supplement: S1 File — (DOCX) [file pone.0233950.s007.docx]

**S1 File. Open-ended questionnaire distributed at initial stakeholder meeting**

## Total Systems Effectiveness (TSE)

## Criteria Prioritisation

**Question 1:**

Please specify your organisation/department (e.g. ITAGI, EPI, WHO CO, etc)

**Question 2:**

In your opinion, which are the top 5 most important criteria to consider when prioritising between vaccine products? Please rank the criteria in order of importance (1=most important, 5=least important).

| Criterion | Rank  (1=most important, 5=least important) |
| --- | --- |
|  |  |
|  |  |
|  |  |
|  |  |
|  |  |
