# Supplementary material for: ‘It takes two to tango’: Bridging the gap between country need and vaccine product innovation
Source: PLoS One. 2020 Jun 10;15(6):e0233950. doi: 10.1371/journal.pone.0233950 (PMC7286512; doi:10.1371/journal.pone.0233950)
Supplement: S2 File — (DOCX) [file pone.0233950.s008.docx]

**S2 File. Feedback form distributed at stakeholder research and development meeting**

**Description of self-assessment form**

You are receiving this self-assessment form as you are a participant at the Total Systems Effectiveness (TSE) for Research and Development workshop being held on Wednesday, 1 August, 2018 in Nonthaburi, Thailand. The meeting is co-organized by the World Health Organization (WHO) and the Health Intervention and Technology Assessment Program (HITAP). The purpose of this form is to inform our understanding of how health technology assessment can inform R&D.

**What do we want from you?**

We are keen to learn from you and urge you to respond to the five questions based on your personal experience. Please note that your responses need not reflect your organization’s position. You may respond in any way you wish, using anecdotes or vignettes, for example. Please provide evidence where possible. There is no space limitation and you may complete the form in the time allotted.

Your responses to this form are confidential and will only be seen by the TSE team. The information provided will be analysed and included in a report or paper anonymously.

**What should you do after completing the form?**

Kindly return your completed self-assessment form to HITAP staff at the event.

| **Questions** |
| --- |

1. **Please tell us about the type of organization you work at:**

□ Government □ Private sector □ International Organization

1. **Which country do you work in:**

□ Thailand □ Other

1. **Do you think that TSE which implies using health technology assessment to inform R&D can be used in the country you work in?**

□ Never □ Yes, in 10 years or more □ Yes, within 10 years

Please explain your response.

|  |
| --- |

1. **In your opinion, what are the obstacles in introducing TSE in your country for the government and the private sectors?**

| Government |
| --- |
| Private sector |

1. **In your opinion, what is the type of support needed for TSE to be used in your country, from the government, private and other sectors?**

| Government |
| --- |
| Private sector |
| Other sectors (please specify) |

1. **Do you have any other comments?**

|  |
| --- |

**--- End ---**
